# Correlation between global longitudinal strain and SYNTAX score in coronary artery disease evaluation

**DOI:** 10.1186/s43044-020-00064-2

**Published:** 2020-05-15

**Authors:** Gomaa Abdelrazek, Alaa Yassin, Khaled Elkhashab

**Affiliations:** Fayoum University, Fayoum, Egypt

**Keywords:** Chronic coronary syndrome, Two-dimensional speckle tracking, Global longitudinal strain, SYNTAX score

## Abstract

**Background:**

Speckle tracking echocardiography may allow the evaluation of myocardial systolic and diastolic dynamics across different physiologic and pathologic conditions beyond traditional echocardiographic techniques. The use of STE longitudinal strain in identification and risk stratification of CAD has good reproducibility and accuracy. The study aims to detect the relationship between SYNTAX score and global longitudinal peak systolic strain (GLPSS) in patients undergoing coronary angiography, with no history of myocardial infarction.

**Results:**

The study included 70 symptomatic patients suspected to have chronic coronary syndrome aging 20–80 years (excluding those with significant structural heart disease). All patients underwent clinical evaluation, surface ECG, laboratory assessment, transthoracic echocardiographic (TTE), color TDI tracings, two-dimensional speckle tracking, and conventional coronary angiography with SYNTAX score calculation. Patients were divided into 3 groups based on the results of the coronary angiogram: normal CAD on angiogram (*n* = 10, control group), low SS (*n* = 25, SS < 22), and high SS (*n* = 35, SS ≥ 22).

The mean age was 55 ± 9.6 years; 54% were males; two third of patients were hypertensive; 52% were diabetic; high percentage of high syntax score were noticed among males, diabetic patients, and smokers; and also low mean of GLS was noticed among diabetic patients and smokers. There was a statistically significant positive correlation between syntax score and each of LVEDD and LVESD and, on the other hand, statistically significant negative correlation between syntax score and each of E/A, GLS, AP2LS, AP3LS, and AP4LS was noticed. Peak GLS cutoff value of 17.8 and 16.5 showed 84% and 93% sensitivity and 70% and 91% specificity to detect high and low syntax score, respectively.

**Conclusion:**

2D longitudinal strain analysis has incremental diagnostic value over visual assessment during echocardiography in predicting significant coronary artery disease; GLS may offer a potential sensitive tool to detect significant CAD.

## Background

The diagnosis and assessment of chronic coronary syndrome (CCS) involves clinical evaluation, identifying risk factors for atherosclerosis, and specific cardiac investigations such as different stress testing modalities and coronary imaging [1].

Despite the widespread use of imaging and provocative testing, noninvasive identification of patients with CAD remains a clinical challenge; more than half of the patients had normal or non-obstructive CAD on coronary angiography [2].

The diagnosis of CAD using echocardiography mostly depends on the detection of left ventricle (LV) abnormal wall motion and the assessment of LV ejection fraction (EF). However, there are no abnormal LV wall motions at rest in CAD patients without a history of myocardial infarction [3].

The strain values are better than either wall motion or tissue Doppler in the assessment of regional contraction. Also, strain can be used in assessing myocardial viability either at rest or with stress [4].

Significant coronary artery stenosis might cause persistently impaired longitudinal LV function at rest, so 2D-STE is more accurate than conventional 2D echocardiography in evaluating the regional and global myocardial function and assessing infarct size, viability of the infarcted myocardium, and mild changes of myocardial ischemia [5].

Speckle tracking echocardiography is a semi-automated modality so it provides good intraobserver and interobserver reproducibility [6].

The non-invasive quantification of regional myocardial function is an important goal in clinical cardiology. An ultrasonic method that quantifies regional deformation has been introduced based on the principles of “strain” and “strain rate” imaging; it requires a single cardiac cycle for further offline processing and interpretation [7].

STE is a simple, rapid, and accurate method for evaluating the myocardial function, so it is best to assess regional contractile function by measuring peak systolic SR or rate of increase of SR [8].

A good correlation between longitudinal strain and the left ventricular ejection fraction (LVEF) has been shown [8] also; longitudinal strain provides a good quantitative myocardial deformation assessment of each LV segment allowing early detection of systolic dysfunction in patients with preserved LV ejection fraction [9].

The use of STE longitudinal strain can detect and risk-stratify CAD with good accuracy and reproducibility. Strain and SR are homogeneously distributed across the myocardium, so mild changes in either measure suggest myocardial dysfunction. Although strain imaging has a potential role in the diagnosis and management of virtually any myocardial disease, its greatest role is in the detection of ischemic heart disease [10].

The Synergy between Percutaneous Coronary Intervention with Taxus and Cardiac Surgery (SYNTAX) score aims to describe coronary anatomy based on anatomic criteria, such as lesion site and complexity; it can predict clinical outcomes after PCI or CABG in multiple vessel disease (MVD) patients and/or left main coronary artery disease [11].

The SYNTAX score involves complex lesions including bifurcations, chronic total occlusions, thrombus, calcification, and small vessel diffuse disease. The score ranges from 0 to greater than 60 in very complex coronary lesions. Patients with SYNTAX scores of 0 to 22 showed comparable outcomes for CABG and PCI while patients with SYNTAX scores greater than 22 CABG patients showed better outcomes than PCI patients. The high SYNTAX scores indicate complex conditions and represent the greatest risks to patients undergoing PCI. The SYNTAX calculator is online at www.syntaxscore.com [12].

The SYNTAX score is the sum of the points of each individual lesion present in the coronary arteries with > 50% diameter stenosis in vessels > 1.5 mm diameter. The coronary tree is divided into 16 segments according to the AHA classification. The SYNTAX score algorithm then sums each of these features for a total SYNTAX score. A computer algorithm is then queried and the score value is produced [13]. The SYNTAX II score integrates two anatomic and clinical variables to predict 4-year mortality after CABG or PCI. It can accurately predict cardiac mortality and major adverse cardiac events in patients with multivessel specifically unprotected left main coronary disease undergoing PCI [14].

## Methods

This is a prospective cross-sectional study. It was conducted on 70 patients complaining of chest pain who presented to the cardiology outpatient clinic for evaluation of myocardial ischemia.

Enrollment of patients commenced in June 2018 and was completed in April 2019.

Symptomatic patients suspected to have stable coronary artery disease aging 20–80 were included in the study.

### Exclusion criteria

- Patients with acute myocardial infarction or high- risk unstable angina.
- Patients with a history of previous myocardial infarction or previous recent coronary revascularization.
- Patients with resting regional wall motion abnormalities in echocardiography
- Patients with structural heart disease, e.g., significant left ventricular hypertrophy.
- Patients with more than mild valvular lesions.
- Patients with suboptimal echogenic window.
- Patients with persistent arrhythmia or frequent extra systoles.

Each patient included in the study has been subjected to the following:

- Careful history taking and thorough physical examination.
- 12-lead surface ECG.
- Transthoracic echocardiography (TTE): Conventional 2D echocardiography was done for each patient then offline 2D-STE analysis was done for the digitally stored images to obtain the longitudinal deformation parameters at rest. Examination was done by using an ultrasound system (Philips EPIQ 7), with a frequency of 1.5–4.3 MHz transducer and high frame rate (60–90 frames/s); LV volumes were traced manually at end-diastole and end-systole in apical four- and two-chamber views using the modified biplane Simpson’s method. The mitral inflow velocity curves were measured using pulsed-wave Doppler in the apical position at the tips of the mitral valve leaflets, and the E/A-ratio was calculated. Peak velocity of early (E) and atrial (A) diastolic filling and deceleration time of the E-wave (DT) were measured. Color TDI tracings were used to measure the E/e′ after measuring the peak longitudinal early diastolic (e′) velocity from the average of the lateral and septal velocities.
- Two-dimensional speckle tracking echocardiography: Three standard apical views were obtained at rest according to the American Society of Echocardiography recommendation. Gain, depth, and sector width were adjusted properly in order to set the frame rate 60 to 80 frames/s. Well-defined cardiac cycle was acquired for each view and stored digitally for offline analysis using the Cardiac Motion Quantification (CMQ) feature on the Q lab 9 software (Philips ultrasound). The end-systole was defined first by defining the aortic valve closure (AVC) as seen in the apical long-axis view. Then, the region of interest (ROI) was set by identifying three points at the end-diastolic frame; 2 annular points low and inside the myocardium at the level of insertion of the mitral valve and 1 apical point at the endocardial border of the apex. The software automatically tracks the internal (endocardial) border and external (epicardial) border in the subsequent frames in 30 s. Each ROI is divided equally by the software into three segments (basal, mid, and apical), in addition to a shared apical cap. Tracking was accepted or rejected and manually corrected by the operator when needed. Numerical and graphical displays of the longitudinal strain were automatically generated for all segments from each view. The same procedure was followed with the apical 4- and apical 2-chamber views, using the same sequence. Finally, after completion of the analysis of the three apical views, the software generates a 17 segment bull’s eye display of the peak longitudinal systolic strain with automatic calculation of the mean global longitudinal strain (GLS).
- Coronary angiography: All patients underwent coronary angiography on a Siemens Axiom Artis coronary angiographic system (Siemens, Erlangen, Germany) within 1 month of the echocardiography. Selective left and right coronary angiography in multiple views in order to avoid side branch overlaps and foreshortening of the relevant coronary stenosis.
- SYNTAX I score calculation: All images were evaluated by an experienced operator who was blinded to the results of STE. The SYNTAX score was derived from the summation of the individual scores for each separate lesion defined as ≥ 50% luminal obstruction in vessels ≥ 1.5 mm. The SYNTAX scores were calculated for all patients using dedicated software (available at http://www.syntaxscore.com/calc/start.htm). Thereafter, the patients were divided into 3 groups based on the presence and/or the severity of coronary artery disease (CAD): no CAD on angiogram (*n* = 10, control group), low SS (*n* = 25, SS < 22), and high SS (*n* = 35, SS ≥ 22).

### Statistical analysis

The collected data were organized, tabulated, and statistically analyzed using SPSS software statistical computer package version 18 (SPSS Inc., USA). For quantitative data, the mean and standard deviation (SD were calculated. Independent *t* test or one-way ANOVA was used in comparing between any two groups or three groups, respectively. Qualitative data were presented as number and percentages, and chi-square (*χ*^2^) was used as a test of significance. Pearson’s correlation was run to identify the relation of SYNTAX score and GLS with study parameters. The receive operating characteristic (ROC) curve was used to determine the discrimination value of GLS for low and high SYNTAX score and to define optimal cut-points for sensitivity and specificity. For the interpretation of the results of tests of significance, significance was adopted at *p* ≤ 0.05.

### Ethics approval and consent to participate

The study was approved by our Institutional ethics committee and an informed written consent was obtained from each patient according to the rules of the Local Ethical Committee of our Faculty (committee reference number: not applicable).

## Results

There were no significant statistical differences between CAD and control groups as regard age, sex, DM, HTN, dyslipidemia, smoking, LVEDD, LVESD, and EF (*p* > 0.05).

The CAD group has significantly lower AP4 longitudinal strain compared to the control group (13.9 ± 3.5 vs. 19.7 ± 2.9, *p* < 0.001), lower AP3 longitudinal strain compared to the control group (14.3 ± 3.4 vs. 18.2 ± 2.8, *p* < 0.001), lower AP2 longitudinal strain compared to the control group (14.1 ± 4 vs. 17.7 ± 3.3, *p* < 0.01), and lower global longitudinal strain compared to the control group (14.03 ± 2.3 vs. 18.5 ± 1.4, *p* < 0.001) (Table 1).

**Table 1:** Comparisons of SYNTAX score categories with echo findings

Variables	Normal (*n* = 10)	Low (*n* = 25)	High (*n* = 35)	*p* value
	Mean ± SD	Mean ± SD	Mean ± SD	
**Age (years)**	51.4 ± 6.2	58.9 ± 9.8	54.2 ± 9.6	0.06
**LVEDD**	41.7 ± 6.1	41.4 ± 5.3	**47.6 ± 5.6**	**< 0.001**
**LVESD**	28.2 ± 5.1	28.04 ± 3.2	**33.2 ± 4.8**	**< 0.001**
**EF**	61 ± 5.6	60.5 ± 5.01	58.4 ± 4.3	0.1
**IVS**	0.93 ± 0.13	0.91 ± 0.12	0.95 ± 0.09	0.2
**PW**	0.92 ± 0.12	0.92 ± 0.012	0.96 ± 0.09	0.2
**E/A**	1.1 ± 0.33	0.84 ± 0.29	**0.82 ± 0.23**	**0.04**
**DT**	157.9 ± 21.8	140.1 ± 25.5	146.4 ± 28.2	0.2
**E/e**	8 ± 0.67	8.5 ± 1.1	8.2 ± 1.4	0.5
**GLS**	18.5 ± 1.4	15.7 ± 2.1	**12.9 ± 1.6**	**< 0.001**
**AP2LS**	17.6 ± 3.3	16.6 ± 3.6	**12.3 ± 3.2**	**< 0.001**
**AP3LS**	18.2 ± 2.8	15.4 ± 4.04	**13.4 ± 2.7**	**< 0.001**
**AP4LS**	19.7 ± 2.9	15.4 ± 3.8	**12.9 ± 2.9**	**< 0.001**
**Creatinine**	1.02 ± 0.3	1 ± 0.2	1.1 ± 0.3	0.5

*LVEDD* left ventricle end-diastolic dimension, *LVESD* left ventricle end-systolic dimension, *EF* ejection fraction, *IVS* inter-ventricular septum, *PW* posterior wall, *DT* deceleration time, *GLS* global longitudinal strain, *AP2LS* apical two longitudinal strain, *AP3LS* apical three longitudinal strain, *AP4LS* apical four longitudinal strain

Higher SYNTAX score significantly correlated with LVEDD and LVESD (*p* < 0.001), E/A ratio (*p* < 0.04), AP2L strain, AP3L strain, AP4L strain, and GLS (*p* < 0.001) (Table 1).

GLS significantly correlated with serum creatinine (*p* = 0.03), LVEDD 9 (*p* = 0.02), LVESD (*p* = 0.004), E/A ratio (*p* = 0.008), AP2L strain, AP3L strain, AP4L strain, GLS, and SYNTAX score (*p* < 0.001) (Table 2).

**Table 2:** Correlation between SYNTAX score and GLS with all study variables among study groups

Variables	GLS	
	*r*	*p* value
**Age (years)**	0.02	0.9
**LVEDD**	**− 0.28**	**0.02**
**LVESD**	**− 0.34**	**0.004**
**EF**	**0.25**	**0.04**
**IVS**	**−** 0.006	0.9
**PW**	**−** 0.07	0.6
**E/A**	**0.32**	**0.008**
**DT**	0.08	0.5
**E/e**	**−** 0.07	0.6
**AP2LS**	**0.72**	**0.001**
**AP3LS**	**0.68**	**0.001**
**AP4LS**	**0.72**	**0.001**
**Creatinine**	**− 0.26**	**0.03**
**SYNTAX score**	**− 0.83**	**0.001**

*LVEDD* left ventricle end-diastolic dimension, *LVESD* left ventricle end-systolic dimension, *EF* ejection fraction, *IVS* inter-ventricular septum, *PW* posterior wall, *DT* deceleration time, *GLS* global longitudinal strain, *AP2LS* apical two longitudinal strain, *AP3LS* apical three longitudinal strain, *AP4LS* apical four longitudinal strain

**Fig. 1:**
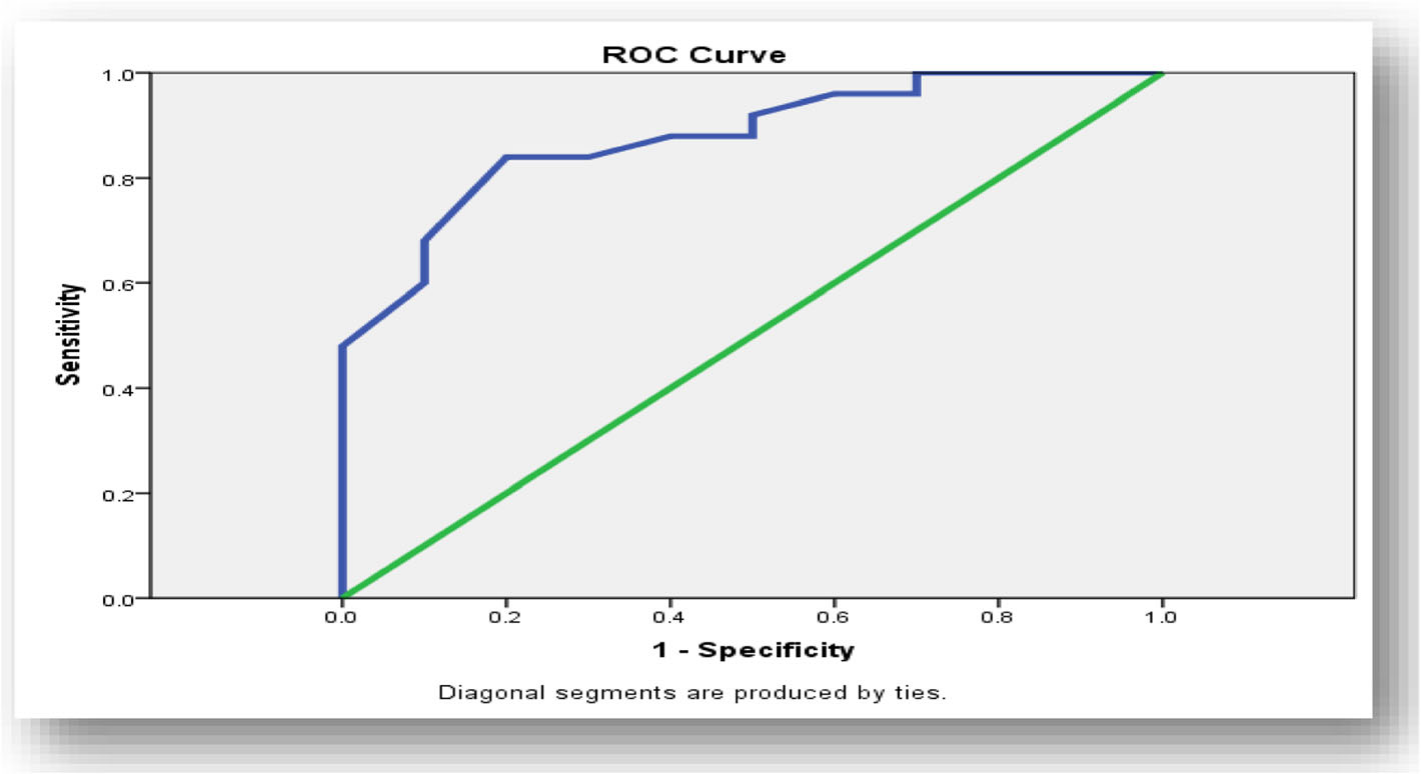
ROC curve for low SYNTAX score

**Fig. 2:**
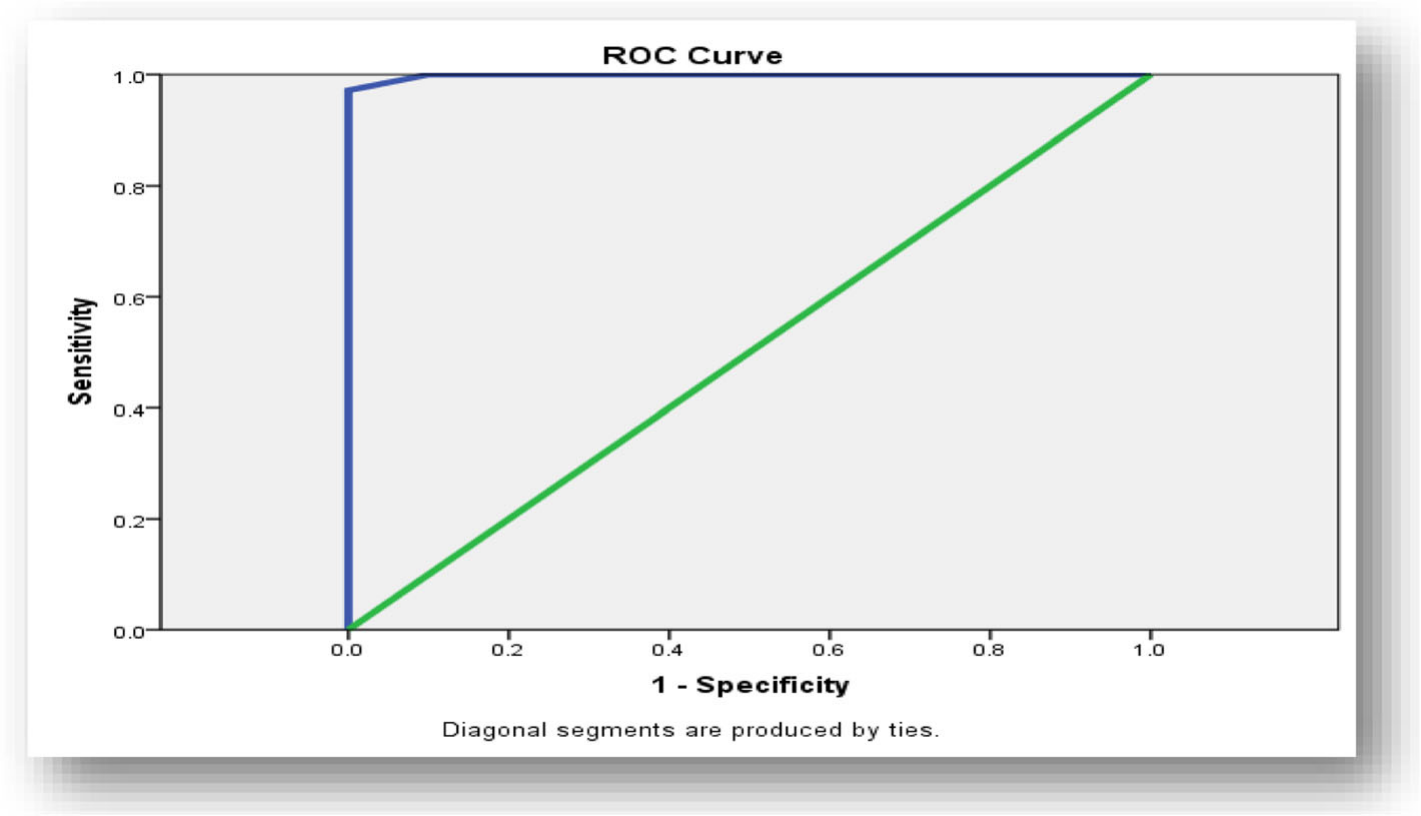
ROC curve for high SYNTAX score

Using GLS cutoff value of 17.8% predicted low SYNTAX score with 84% sensitivity and 70% specificity (Fig. 1). On the other hand, using GLS cutoff value of 15.9% predicted high SYNTAX score with 97.1% sensitivity and 90% specificity (Fig. 2).

## Discussion

Echocardiography is an important cardiac imaging tool in patients with suspected cardiac disease. However, conventional echocardiography has a limited value in the diagnosis and risk stratification of suspected CAD patients as most of these patients with no previous myocardial infarction or myocardial stunning have no wall motion abnormalities at rest. So, finding another resting module to diagnose and predict the severity of CAD would be very beneficial [15].

Speckle tracking echocardiography (STE) is a semi-automated software that allows fast, quantitative, and angle-independent assessment of the three components of myocardial deformation, with great feasibility and reproducibility particularly of the longitudinal one. Several clinical studies confirmed the feasibility of STE-derived longitudinal strain analysis as an adjunctive method for CAD detection [16].

In this prospective study, we studied 70 patients aged 20–80 and collected from an outpatient cardiology clinic; echocardiography (including both conventional and STE) and coronary angiography were done for each patient, and then data was collected and analyzed.

We could identify a cutoff value of GLS with high sensitivity and specificity to detect a high SYNTAX score.

Biering-Sørensen et al. [17], Gaibazzi et al. [18], and Billehaug et al. [19] showed that GLS is significantly lower in patients with obstructive CAD (at least one stenosis > 50% or ≥ 70% luminal area reduction) when compared with patients with non-obstructive CAD, and our study showed similar results. And they reported that GLS values at rest have moderate diagnostic accuracy in predicting significant CAD while in our study, it had high diagnostic accuracy, as our results revealed that GLS ⩽ 16.5 can predict significant coronary artery stenosis, with sensitivity 93% and specificity 91%, while Biering-Sørensen et al. [17] showed that GLS ⩽ − 18.4% can predict significant coronary stenosis (> 70%), with sensitivity 74% and specificity 58%. Similarly, Gaibazzi et al. [18] showed that GLS ⩽ − 20.7 may predict significant coronary stenosis (> 50%), with sensitivity 81.6% and specificity 84.9%.

In Billehaug et al. [19], GLS measurements have moderate diagnostic accuracy in predicting significant CAD in patients presenting with chest pain. They showed that GLS cutoff value for prediction of CAD varied between − 17.4 and − 19.7% with sensitivity from 51 to 81% and specificity between 58 and 81%. The effect of diastolic function and afterload on GLS may explain this finding.

Our study showed that GLS decreased incrementally with increasing SYNTAX score which indicates increasing severity of CAD. Vrettos et al. [20] showed similar results when they studied 71 patients and reported that GLS values were inversely correlated to SYNTAX score values. And they showed that the GLS optimal cutoff value to detect patients with high SYNTAX score was − 13.95 (sensitivity = 71%, specificity = 90%, *p* < 0.001).

There was a positive correlation between high SYNTAX score and being diabetic (*p* = 0.007); this was also noted in Srinivasan et al. [21] where they observed that patients with 5–10 years of diabetes mellitus have a significant increase in the mean SYNTAX score (*p* = 0.019) when compared to those with less diabetes duration.

Another positive correlation was present between SYNTAX score and smoking (*p* = 0.001); this was found in several studies, among them El Kersh et al. [22], where a statistically significant correlation between age, hypertension, diabetes mellitus, dyslipidemia, and smoking with SYNTAX score was noticed (*p* < 0.05).

El-Sayed et al. [23] found that high SYNTAX score patients have higher E/A ratio and lower deceleration time (DT) when compared with the low SYNTAX score patients (*p* = 0.016 and *p* = 0.046, respectively) while our study did not reach these results as the E/A ratio was lower in the high SYNTAX group (*p* = 0.04).

In our study, mean GLS was lower in diabetic patients 13.7 ± 2.4 than non-diabetics 15.8 ± 2.7 (*p* = 0.001), and this was similar to the results of Elgohary et al. [24] who compared results of patients with controlled and uncontrolled diabetes and found a significant statistical difference in GLS, age, diabetic duration, 2HPP blood sugar level, and E/é ratio in patients with controlled DM compared to uncontrolled DM. Another study by Wierzbowska-Drabik et al. [25] showed that during DSE global and regional LV peak, systolic longitudinal strain revealed lower values in DM patients when compared to non-diabetics: 14.5 ± 3.6% vs. 17.4 ± 4.0% at rest; *p* = 0.0001.

On the other hand, while we found that smokers had significantly lower mean GLS (*p* = 0.002), Farsalinos et al. [26] showed no significant changes in GLS were reported when they studied myocardial function in young and healthy heavy smokers.

Our study results showed a good correlation between left ventricle EF and GLS (*r* = 0.25; *p* = 0.04), and Benyounes et al. [27] reported similar results when they approved that two-dimensional GLS can predict LVEF (*r* = − 0.53; *p* < 0.001). Also, Lima et al. [28] concluded that left ventricle EF and GLS showed a powerful correlation (*r* = 0.95; *r*^2^ = 0.89; *p* < 0.001), especially in patients with LV systolic dysfunction than those with normal LVEF.

## Conclusion

- Normal left ventricular wall motion at rest cannot exclude significant CAD.
- GLS has a significant correlation with the complexity of CAD as quantified by the widely used scoring system, SYNTAX score.
- Resting 2D-STE-derived global longitudinal strain (GLS) can predict significant CAD with high sensitivity of 84 and 93% for the detection of low and high SYNTAX score, respectively.

### Clinical applications and recommendations

The present study recommends that adding STE to the standard echocardiography protocol for evaluation of myocardial ischemia in patients with chronic coronary artery disease and using the found cutoff values of GLS can predict patients likely to have more severe and complex CAD and might benefit from invasive coronary angiography and revascularization, but these values should be tested in larger population studies.

### Limitations

- Relatively low number of studied patients and single-center study.
- Excluding patients with wall motion abnormalities at baseline echocardiogram.
- Strain and strain rate are influenced by left ventricular mass, various hemodynamic variables, and software platform type, in addition to myocardial ischemia. All these factors could be potential confounders.

## Data Availability

The data sets used and/or analyzed during the current study available from the corresponding author on reasonable request.

## Abbreviations

2D-STE: Two-dimensional speckle tracking echocardiography
AHA: American Heart Association
AP2LS: Apical two longitudinal strain
AP3LS: Apical three longitudinal strain
AP4LS: Apical four longitudinal strain
CABG: Coronary artery bypass grafting
CAD: Coronary artery disease
DM: Diabetes mellitus
DSE: Dobutamine stress echocardiography
DT: Deceleration time
ECG: Electrocardiogram
EF: Ejection fraction
ESC: European Society of Cardiology
GLPSS: Global longitudinal peak systolic strain
GLS: Global longitudinal strain
HTN: Hypertension
IHD: Ischemic heart disease
IVS: Inter-ventricular septum
LBBB: Left bundle branch block
LV: Left ventricle
LVEDD: Left ventricle end-diastolic dimension
LVEF: Left ventricular ejection fraction
LVESD: Left ventricle end-systolic dimension
LVH: Left ventricular hypertrophy
MI: Myocardial infarction
PCI: Percutaneous coronary intervention
PW: Posterior wall
PVD: Peripheral vascular disease
ROC: Receiver operating characteristic
ROI: Region of interest
SD: Standard deviation
SPSS: Statistical Package of Social Science
SR: Strain rate
TDI: Tissue Doppler imaging
WMA: Wall motion abnormalities
